# Evaluation of the potential denture covering area in buccal shelf with intraoral scanner

**DOI:** 10.1016/j.jds.2022.04.010

**Published:** 2022-04-26

**Authors:** Worachate Romalee, Matana Kettratad, Tran Thi Ngoc Trang, Ding-Han Wang, Jia-En Chen, Ming-Lun Hsu

**Affiliations:** a College of Dentistry, National Yang Ming Chiao Tung University, Taipei, Taiwan; b Department of Community Dentistry & Gerodontology, Faculty of Dentistry, Thammasat University, Pathum Thani, Thailand; c Medical 3D Printing Center, Tri-Service General Hospital, National Defense Medical Center, Taipei, Taiwan; d Department of Biomedical Engineering, National Defense Medical Center, Taipei, Taiwan

**Keywords:** Intraoral scan, Dentures, Buccal shelf, Denture base adaptation

## Abstract

**Background/purpose:**

The characteristic of soft tissue changes in buccal shelf area during function is unclear. This study aimed for evaluating the potential denture covering area in buccal shelf area in different ranges of mouth opening by a digital approach.

**Materials and methods:**

Nineteen qualified dentate participants were enrolled. An intraoral scanner was used to record soft tissue in buccal shelf area in different ranges of mandibular opening, which were maximum intercuspation, 10 mm, 20 mm, and 30 mm of interincisal distance. The experiment was performed by two examiners. The common area of each range was generated within the clinically acceptable denture adaptation range, which was represented as the potential denture covering area. Data were statistically analyzed using descriptive statistic, one-way repeated measure ANOVA, intraclass correlation coefficient (ICC), and the Pearson correlation test.

**Results:**

Trends of the mean distance of the potential denture covering area increased from the frenum area to the second premolar and the first molar area then decreased in the second molar area, along with the ranges of mouth opening increased. The distance in the second molar area had drastic percentage changes during the mouth opening. The mean distances changed significantly when the mouth opening increased (*P* < 0.001). All ICC values of intra-, inter-rater reliability indicated good to excellent reliability. The correlation between the results from two examiners was strong (*P* < 0.001).

**Conclusion:**

The characteristic of the denture covering area posteriorly to the first molar area is inversely proportional in length to ranges of mouth opening.

## Introduction

For tissue supported or tooth-tissue supported dentures with distal ended, a denture flange should be totally covered the area of buccal shelf to maximize the tissue-denture contact area without interfering the function of muscles, which would positively affect the retention, stability, and support of the denture.^1^, ^2^, ^3^ However, determining and recording soft tissue in buccal shelf using digital technology has not yet been fully investigated especially a characteristic of soft tissue changes at different ranges of jaw opening and the reliability of the scanning method.

Buccal Shelf is the bony structure bilaterally located on the posterior lateral area of mandible. It is bounded medially by alveolar bone, laterally by external oblique ridge, anteriorly by buccal frenum, and posteriorly by retromolar pad.^1^^,^^2^ Because of a strong cortical bone and buccinator muscle attachment around this area, buccal shelf can stand with the higher occlusal force and does not prone to resorb when compared to other adjacent structures.^3^ Accordingly, it is considered as a primary stress bearing area for a denture.^4^ Overextended borders could affect the retention and stability of the dentures as well as causing traumatic lesions. On the other hand, underextended borders also could affect the retention and support of the dentures. According to two systematic reviews, the most common complaint after delivering removable denture and complete denture are loss of retention and ulcer,^5^^,^^6^ which mostly occurred in mandibular posterior area.^6^ The proper extension of the denture borders is considered as one of the preferable factors when treating denture worn patients. However, the method to define the border of denture covering areas, border molding,^7^ perceived by novice dentists as an advanced skill and requiring clinical experience.^8^

Digital technology has massive influences on daily dental practices and research. Many dental techniques were proposed as protocols for the removable denture and complete denture fabrication.^9^^,^^10^ Unfortunately, the current digital technology still does not permit the recording of peripheral boundaries in a truly functional state.^9^ On the other hand, digital equipment such as intraoral scanner still can be used for recording adjacent soft tissue to those boundaries i.e., attached gingiva^11^, ^12^, ^13^ and buccal shelf^14^ with the comparable result when compare with the conventional methods.

The clinical acceptable range of denture base adaptation was proposed in the recent systematic reviews. Within 0.3 mm deviation from the static state of soft tissue in denture bearing area, the denture adaptation was considered as clinical acceptable.^15^ According to the previous studies, the deviation could be gaps between the intaglio surface of the denture base and gypsum cast or the sunk soft tissue after occlusal forced applied.^16^, ^17^, ^18^

The purpose of this study was to evaluate the characteristic of the potential denture covering area in the buccal shelf area at different ranges of mouth opening by using intraoral scanner. In addition, the reliability of the soft tissue scanning in this area was evaluated.

## Materials and methods

This study was conducted in the international education center of Digital Dentistry, College of Dentistry, National Yang Ming Chiao Tung University, Taiwan. Ethical approval was approved by the institutional review board (IRB) of National Yang Ming Chiao Tung University (Approval number: YM110113F). All participants were explained about the significance and content of this study before signing the informed consent. Participants with temporomandibular disorder or limited mouth opening, which was less than 30 mm; and participants who received orthodontics treatment with tooth extraction on the investigated areas were excluded. Subjects without missing teeth in the investigated area and without exclusion criteria were included.

In this study, 10 men and 9 women (mean age: 27.3 ± 5.1 years, range: 20–36 years) were enrolled. It was sufficient for one-way repeated measure analysis of variance, which needed at least 9 participants, as estimated using a statistical analysis software (GPower, v3.1.9.7; University of Duesseldorf, Duesseldorf, Germany) under the circumstances of α = 0.05 and power = 0.80. Nineteen participants were also sufficient for testing intraclass correlation coefficient (ICC), which needed at least 10 participants, as calculated based on the guide to determination of sample size requirements for estimating the value of ICC to achieve 0.8 power of significant difference detection of expected ICC value 0.7 with α = 0.05, 2 examiners and 3 times of experiment repetition.^19^^,^^20^

Data acquisition was performed by two examiners who have experienced using an intraoral scanner. The intraoral scanner (TRIOS 3; 3Shape A/S, Copenhagen, Denmark) was used to record soft tissue in the left buccal shelf area in different ranges of mandibular opening at maximum intercuspation position (MIP), 10 mm, 20 mm, and 30 mm of interincisal distance, respectively. AI mode of the IOS was turned off. All participants were scanned 3 times by each operator. Before scanning vinylpolysiloxane impression material (Elite HD + Putty Soft; Zhermack S.p.A, Badia Polesine (RO), Italy) was used for making anterior occlusal jigs. Cheek retractors (Cheek retractor TypeB; DiaDent, British Columbia, Canada) were used for retracting lips and cheeks during scanning. Resin blocks with different sizes (10 mm, 20 mm, and 30 mm) were printed by using 3D printing material (Model-S2 Resin; PrintIn3D DigiTech, Taoyuan, Taiwan). The Silicone jigs and 3D printed resin blocks were used for indicating the ranges of mouth opening.

During scanning, all participants sat in upright position. The scanning method had 2 steps. The initial step was to scan teeth to use them as scanning references: beginning from canine tooth, IOS was used to scan in horizontal direction to the second molar tooth. The second step was to scan soft tissue in buccal shelf area: beginning from soft tissue in the second molar area, IOS was used to scan soft tissue outward by still overlapping the scanning references. The IOS tip was kept close to the teeth as much as possible to minimize buccal mucosa stretching. All the scanned files were in STL format.

**Figure 1:**
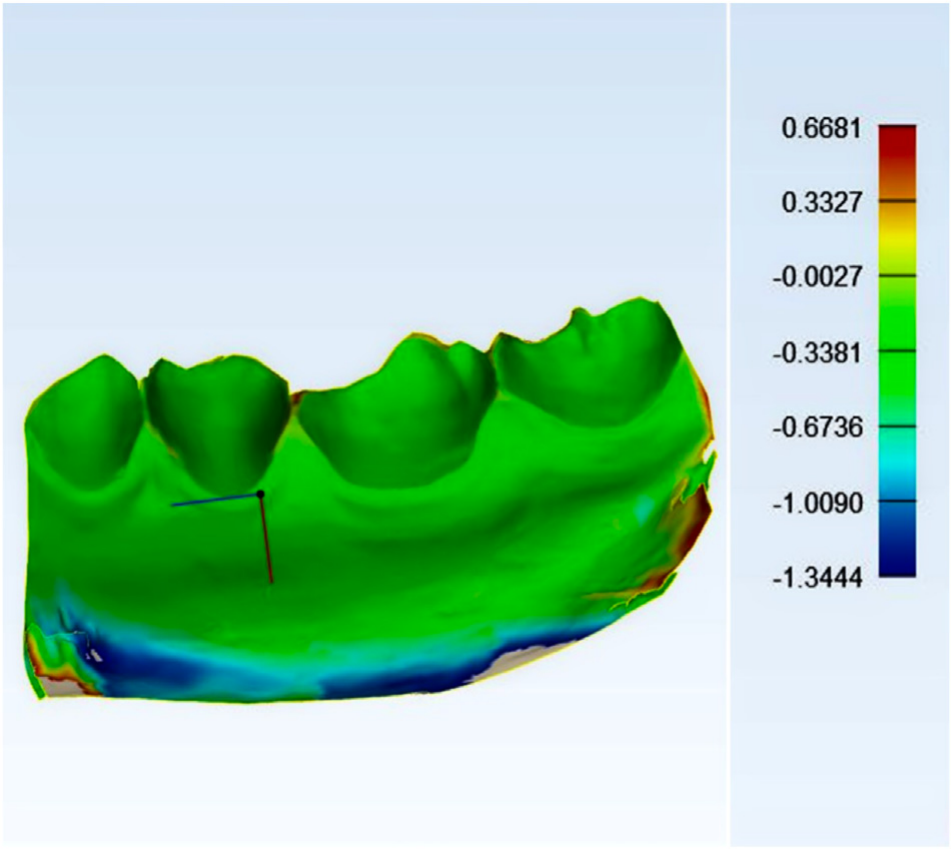
The comparison results between reference model and compared model (10 mm) without adjustment of the comparison range. The range of comparison was generated automatically by the software. The comparison results were displayed on the reference model.

**Figure 2:**
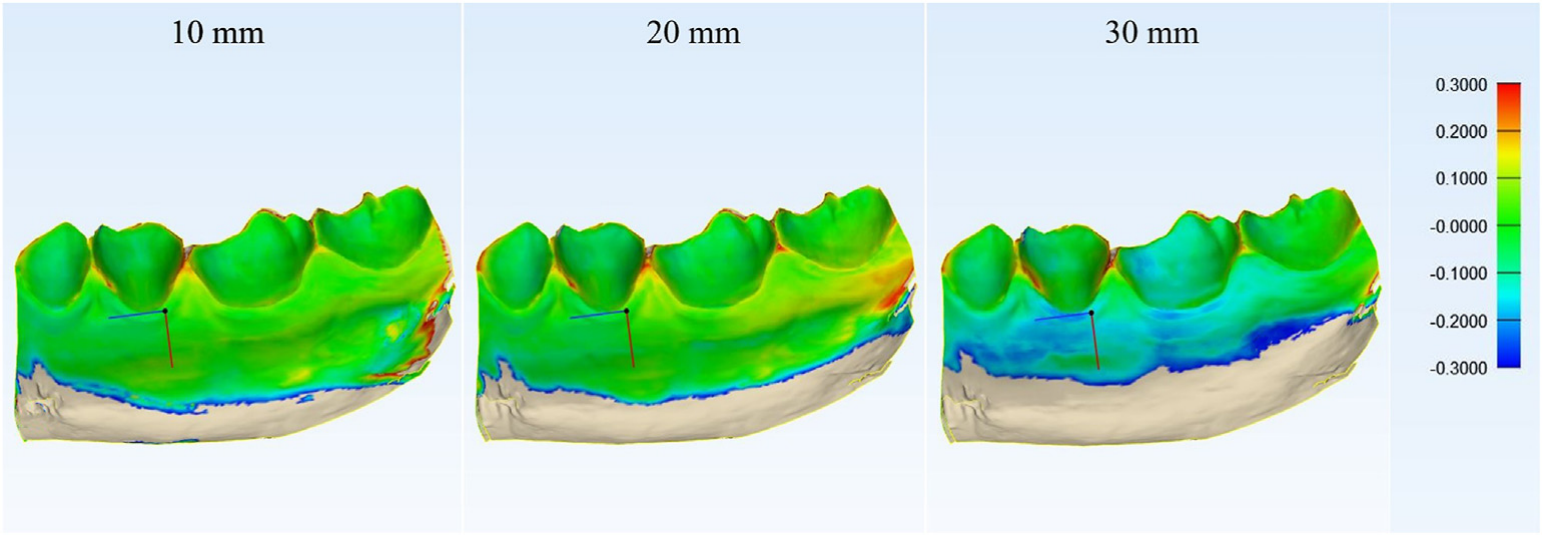
The comparison results between reference model and compared models (10, 20, 30 mm) after the comparison range was adjusted to −0.3 mm–0.3 mm. The comparison results within the range of clinically acceptable denture base adaptation were displayed as the same colors as in the right bar. The differences apart from the acceptable range were showed as the reference models color (stone color).

Scanned files from the intraoral scanner were imported into 3D analysis software (Materialise 3-matic, v14.0; Materialise, Leuven, Belgium). The MIP files of each participant were used as reference models, while the scanned files in other different positions were used as compared models. The reference models and the compared models were aligned and superimposed by semi-auto registration method and using teeth as superimposed references. 3D comparison was performed within the range from −0.3 mm to 0.3 mm according to the range of clinically acceptable denture base adaptation (0.3 mm).^15^ Common areas between the reference and compared models were shown on reference models and considered as a potential denture covering area (Figure 1, Figure 2).

**Figure 3:**
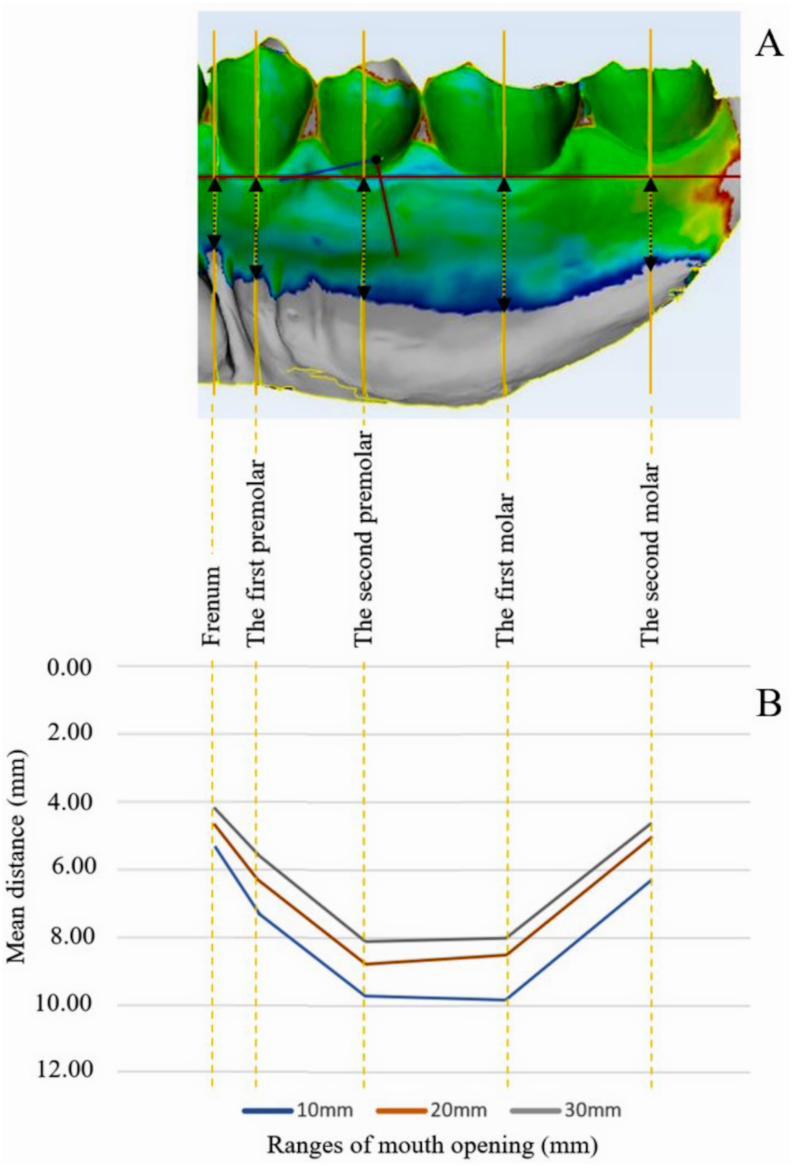
The reference lines and points for measurement and mean distances results. The Fig. 3A shows the reference horizontal line (red), the direction lines of measurement (yellow) that pass-through reference points, including frenum, the first premolar, the second premolar, the first molar, and the second molar, and the measured distances (black arrow). The Fig. 3B shows the connected mean distance lines between each reference point at different ranges of mouth opening. This picture could represent the character of denture borders in the buccal shelf area.

Reference horizontal lines were drawn from the lowest part of the first premolar and the first molar in each reference model. The reference points consisted of the highest part of buccal frenum, the lowest part of first premolar, second premolar, first molar, second molar areas. The distances of the potential denture covering area were measured perpendicularly from the reference horizontal plane of the models in each reference point to the border of the common areas (Fig. 3A).

The acquired data were analyzed by a statistical software program (SPSS Statistics for windows, v21.0; IBM, New York, NY, USA). Mean distances and standard deviations of these measured data in each reference point with different ranges of mouth opening were calculated. The mean distances of the potential denture covering area were compared between 10 mm, 20 mm, and 30 mm by One-way repeated measure analysis of variance. When the mean distances were significantly different, the Bonferroni test was performed (α = 0.05). The intra- and inter-rater reliability were calculated by ICC with α = 0.05, power of 0.80 (1−β error probability). The Pearson correlation was used to evaluate the correlation between the results from two examiners.

## Results

The descriptive results of the potential denture covering areas at different reference points at different ranges of mouth opening are listed in Table 1. The results showed that the mean distances decreased continuously in every reference point while the ranges of mouth opening increased. The frenum area was the area that has the lowest mean distances in every range of mandibular function. On the other hand, the first molar area had the longest mean distance at 10 mm mouth opening before changing to the second premolar area at 20 mm and 30 mm of mouth opening.

**Table 1:** Descriptive distance results of each reference point in different ranges of mouth opening.

Ranges of mouth opening (mm)	Reference points	Mean Distance (mm)	Standard Deviation (mm)	Minimum Distance (mm)	Maximum Distance (mm)
10	Frenum	5.37	1.57	2.19	9.31
	The first premolar	7.26	1.50	3.85	11.71
	The second premolar	9.84	1.64	6.46	13.89
	The first molar	9.89	1.83	5.39	13.62
	The second molar	6.24	1.99	1.12	10.68
20	Frenum	4.65	1.39	1.99	9.68
	The first premolar	6.41	1.25	3.38	10.03
	The second premolar	8.80	1.48	6.52	13.03
	The first molar	8.48	1.79	3.57	11.58
	The second molar	5.09	1.66	0.90	9.99
30	Frenum	4.24	1.27	1.20	7.95
	The first premolar	5.67	1.14	2.59	8.63
	The second premolar	8.16	1.56	5.43	12.02
	The first molar	8.07	1.79	3.25	11.54
	The second molar	4.71	1.66	0.73	8.16

The Fig. 3B displays the connected mean distance lines between each reference point at different ranges of mouth opening. The mean distances increased from the frenum area to the second premolar and the first molar area before decreasing in the second molar area.

**Figure 4:**
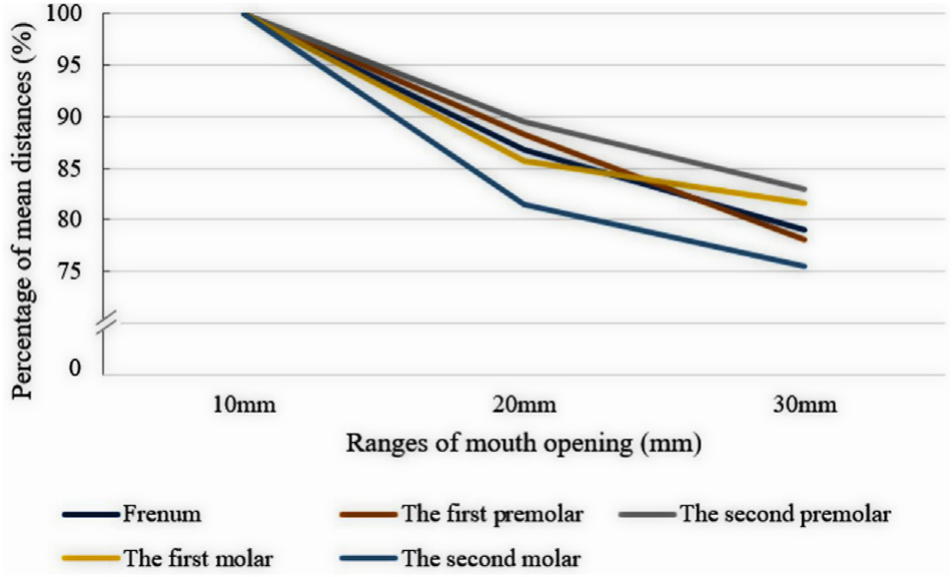
The percentage changes results. The figure shows the percentage changes of mean distances results during mouth opening. The second premolar area had the smallest percentage changes while the second molar area had the highest percentage changes during all ranges of mouth opening.

The percentage changes results are shown in Table 2 and Fig. 4. The second premolar area had the smallest percentage changes when compared with other reference points during the range of mouth opening changed from 10 mm to 20 mm and from 20 mm to 30 mm. In contrast, the second molar area had the highest percentage changes during all ranges of mouth opening. As a result, these indicated that the potential denture covering areas in the second molar area were more sensitive to mouth opening than other area. Furthermore, the figure shows that during the initial phase of mouth opening, from 10 mm to 20 mm, had higher percentage changes when compared to the opening phase from 20 mm to 30 mm in every reference point.

**Table 2:** The percentage changes results of the mean distance in different ranges of mouth opening.

Reference points	The percentage of the mean distance in different ranges of mouth opening		
	10 mm	20 mm	30 mm
Frenum	100%	86.75%	79.03%
The first premolar	100%	88.33%	78.10%
The second premolar	100%	89.46%	82.93%
The first molar	100%	85.73%	81.58%
The second molar	100%	81.54%	75.44%

Table 3 shows the results of the One-way repeated measures ANOVA on mean distances of potential denture covering area in different reference points at 10 mm, 20 mm, and 30 mm mouths opening. The mean distances change significantly in every reference point when mouths opened wider (*P* < 0.001), except that there had no significant different change when mouths opened from 20 mm to 30 mm in the first molar area (*P* = 0.092).

**Table 3:** Findings of One-way repeated measures ANOVA on mean distances of potential denture bearing area in different reference points at different ranges of mouths opening.

Reference points	Mean distance (mm) in different ranges of mouth opening			F	*P-value*
	10 (n = 19)	20 (n = 19)	30 (n = 19)		
Frenum	5.37 ± 1.57	4.65 ± 1.39	4.24 ± 1.27	34.78	<0.001
The first premolar	7.26 ± 1.50	6.41 ± 1.25	5.67 ± 1.14	34.77	<0.001
The second premolar	9.84 ± 1.64	8.80 ± 1.48	8.16 ± 1.56	50.37	<0.001
The first molar	9.89 ± 1.83	8.48 ± 1.79^a^	8.07 ± 1.79^a^	41.50	<0.001
The second molar	6.24 ± 1.99	5.09 ± 1.66	4.71 ± 1.66	43.45	<0.001

Same superscript letter above the values indicates group that was not statistically different (*P* > 0.050) after Bonferroni post hoc tests were used.

a *P* = 0.092.

The intraclass correlation coefficient (ICC) values of intra-rater reliability for the first and the second examiner were 0.905 (95% CI 0.886, 0.922) and 0.896 (95% CI 0.875, 0.914), respectively, which indicated good to excellent reliability. The ICC values of inter-rater reliability for overall mouth opening ranges, 10 mm, 20 mm, and 30 mm mouth opening were 0.909 (95% CI 0.862, 0.930), 0.890 (95% CI 0.827, 0.926), 0.893 (95% CI 0.852, 0.921), and 0.909 (95% CI 0.853, 0.940), respectively. All of the ICC values indicated good to excellent reliability.

**Figure 5:**
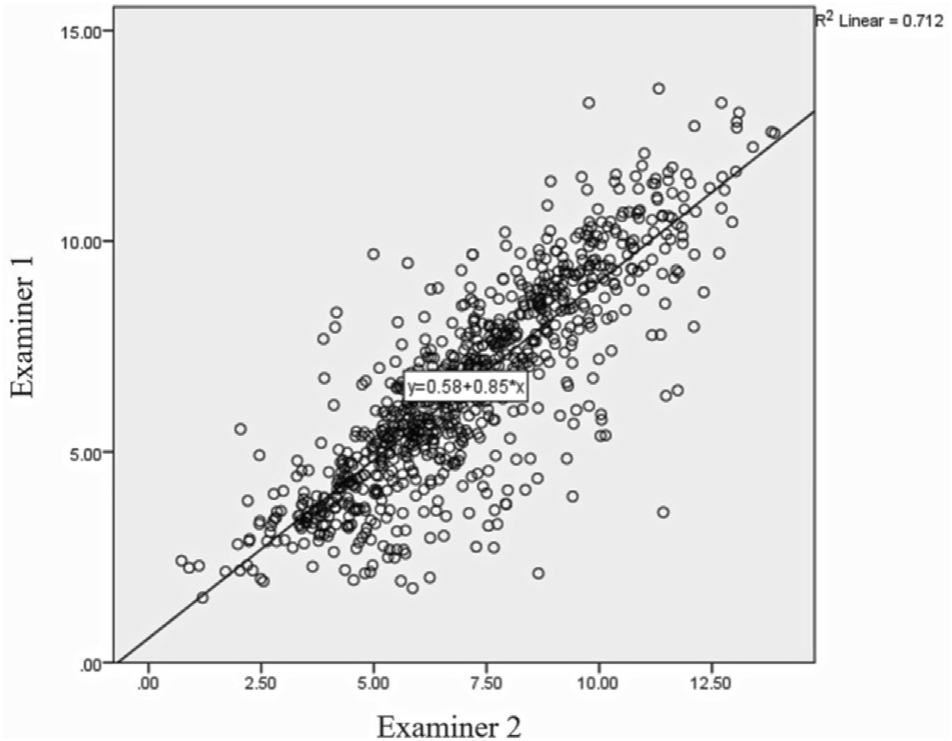
The correlation between the results from two examiners. The correlation indicated a strong linear relationship between the results.

The correlation between the results from two examiners was evaluated by the Pearson correlation. There was a significant correlation with r value = 0.844 (*P* < 0.001). The correlation indicated a very strong linear relationship between the results (Fig. 5).

## Discussion

There was no study provided information about the denture covering area change in the buccal shelf area especially in term of the length of denture flange. Dentists always have been taught to use border molding process to identify the proper border of removable dentures. However, because of different clinical experiences, outcome could be different.^8^ This study provided the result that can be applied to use as a characteristic of denture border in the buccal shelf area. The denture flange should be made short in the frenum area then become longer from the first premolar area to the first molar area. Posterior to the first molar area, the denture border should become shorter to avoid overextension. However, the estimate proportion or reference number could not be suggested in this study since the variety of individual anatomical structures, such as minor buccal frenum, muscle attachment, and the alignment of teeth, still needed to be considered.

From the results of the percentage changes, the potential denture covering area in the second molar area was the most sensitive area to be changed during mouth opening. As a result, the soft tissue movement around this area is prone to be jeopardized by overextended denture borders. Consequently, the buccal mucosa-tongue side wall contact (BTC) point, which is significant for mastication and swallowing, might be interfered.^21^ The length of denture flange in this area should be decreased continuously since the first molar area.

There is a study evaluated the accuracy of soft tissue record in the buccal shelf area.^14^ However, the reliability of the scanning is also important. Since the results from the border molding process could be different from each operator by clinical experience.^8^ On the other hand, there is no study evaluated the reliability of the traditional border molding process. This study has proposed an alternative way to record soft tissue in the buccal shelf area. Accordingly, the reliability tests were performed in this study. The ICC results indicated good to excellent intra- and inter-rater reliability. Moreover, the correlation between the results from two examiners had a very strong linear relationship. As a result, this scanning method provided acceptable reliability. Nonetheless, the comparison with the denture covering area obtained by the traditional border molding process needs further study.

There are several reasons to recruit dentate participants in this study. Lack of soft tissue identities to be references for scanning and 3D superimposition in edentulous participants could be one of the significant reasons.^22^ Furthermore, regarding to residual ridge resorption, the potential denture covering area will be decreased continuously after tooth loss, which has different rate individually.^1^ The results from dentate participants could provide the reference distances of the potential denture covering area before bone resorption. These distances will never be increased but decrease by the time after tooth loss.

There are some limitations of this study need to be considered before applying in clinical practices. Firstly, the results of this study didn't represent truly effect of mouth opening but combined with the effect of the IOS tip that stretched buccal mucosa. This issue could be a suggestion for the IOS development. Decreasing the thickness of the IOS tip might help to reduce the stretching effect. Secondly, denture fabrication processes also need to consider movements in other directions beside a vertical direction when the mouth is opened. Lateral jaw movement, jaw protrusion, jaw retrusion, and even frenum's movement are crucial for denture fabrication. Thirdly, this study was performed with dentate participants to overcome the reference limitation of edentulous scanning. However, edentulous patients might have different result causes by individual residual ridge resorption and references for scanning.

To our knowledge, our study was the first to demonstrate the potential denture covering area by using intraoral scanner with acceptable reliability supported results. The idea of scanning 3D images in different stages then superimpose the images to obtain the common area within a specific range could be adapted for future digital border molding process. However, the limitations, mentioned above, need to be concerned. The future study could be a comparison of the denture covering area between the results of this method and the traditional border molding process. Edentulous participants are necessary for proving the practical of this research but the IOS improvement for scanning edentulous ridge must reach a clinical acceptable level.

## Declaration of competing interest

The authors have no conflicts of interest relevant to this article.
